# Clinical value of methylation testing: a case report of intraventricular schwannomas with associated molecular findings

**DOI:** 10.1093/noajnl/vdaa029

**Published:** 2020-03-26

**Authors:** Justin Z Wang, Neda Pirouzmand, Nazanin Ijad, Carlos Velasquez, Andrew Gao, Shirin Karimi, Yasin Mamatjan, Phedias Diamandis, Gelareh Zadeh, Farshad Nassiri

**Affiliations:** 1 Division of Neurosurgery, Department of Surgery, University Health Network, University of Toronto, Toronto, Ontario, Canada; 2 MacFeeters-Hamilton Neuro-Oncology Program, Princess Margaret Cancer Center, Toronto, Ontario, Canada; 3 Department of Pathology, University Health Network, University of Toronto, Toronto, Ontario, Canada

Schwannomas are benign nerve sheath tumors arising from peripheral, cranial, or autonomic nerves.^1^ Intracranial schwannomas represent 6–8% of all intracranial tumors, the majority located in the cerebellopontine angle. Intraventricular schwannomas are rare with only 31 cases reported.^2^ The wide differential of intraventricular lesions combined with the rarity of intraventricular schwannomas makes diagnosis a challenge, both radiographically and histopathologically. We report on a case of an intraventricular schwannoma and demonstrate how DNA methylation can be used to confirm the unusual diagnosis.

## Case Report

A 44-year-old otherwise healthy Caucasian male with a history of migraines presented with a 1-year history of progressive headaches with occasional nausea and gait instability. Neurological exam including cranial nerve exam was unremarkable. Fundoscopy did not show any evidence of papilledema. Gait was grossly normal. There was no family history of any neoplastic disease or known heritable genetic conditions.

**Figure 1:**
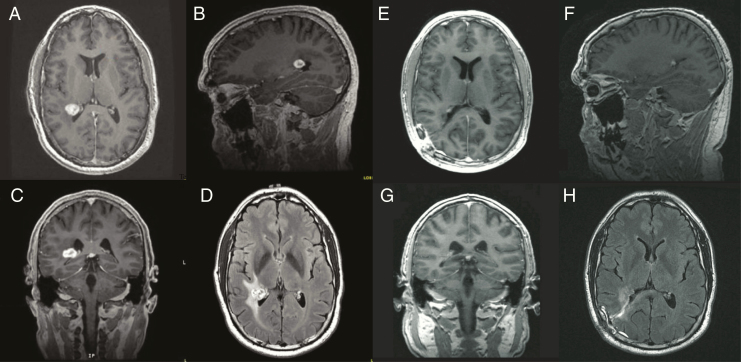
(A–D) Preoperative magnetic resonance imaging (MRI). Enhancing lesion in the lateral ependymal surface of the right lateral trigone measuring 1.7 × 1.4 × 1.2 cm. The medial border is intimately associated with the adjacent choroid plexus. Surrounding vasogenic edema in the periventricular white matter of the right parietal lobe. (A–C) T1-weighted imaging post-gadolinium contrast representative axial, sagittal, and coronal images, respectively. (D) Representative axial T2 FLAIR image demonstrating perilesional edema. (E–H) Postoperative MRI following right parietal craniotomy for tumor resection. A small amount of residual postoperative fluid collection and associated dural thickening adjacent to the craniotomy site shown with the surgical tract leading toward the atrium of the right lateral ventricle. (E–G) Representative axial, sagittal, and coronal T1-weighted imaging post-gadolinium contrast images, respectively. (H) T2 FLAIR image demonstrating a decrease in perilesional edema compared to preoperatively.

Magnetic resonance imaging (MRI) with gadolinium showed a 1.7 × 1.4 × 1.2 cm enhancing tumor centered in the lateral ependymal surface of the right lateral trigone with associated vasogenic edema in the periventricular white matter of the right parietal lobe (Figure 1). The medial border of the lesion was associated with the choroid plexus. Gradient echo sequences demonstrated internal blooming without any associated calcifications on Computed Tomography (CT) of the brain. There was no hydrocephalus or additional areas of ependymal enhancement, and no enlargement of the choroid plexus. CT angiogram did not show any direct vascular involvement. The posterior choroidal arteries were not significantly enlarged and there was no evidence for dilatation of the choroidal or internal cerebral veins, decreasing clinical suspicion of a high flow lesion.

Differential diagnosis for this lesion was primarily neoplastic in nature and included high-grade glioma, ependymoma, subependymoma, meningioma, choroid plexus tumors, metastases, or schwannoma. We trialed a period of surveillance with radiographic and clinical follow-up over several months, without any documented change in lesion character or size. However, given the patient’s escalating symptoms, lack of diagnosis, and concerning vasogenic edema, surgery was ultimately offered.

We performed a right-sided parietal mini-craniotomy and inserted an external ventricular drain (EVD) into the atrium of the right lateral ventricle under stereotactic guidance. The EVD was followed microsurgically into the atrium of the ventricle where the choroid plexus was visualized, and posteriorly, a firm, tan-red-colored mass somewhat adherent to the choroid plexus but not obviously originating from it. We microsurgically devascularized the lesion using bipolar electrocautery and resected it in a piecemeal fashion to achieve a gross total excision. There were no intraoperative complications, and the patient’s postoperative course was unremarkable with a 2-day hospital stay. Postoperative MRI showed near total resection with one small focus of enhancement possibly representing residual tumor. This was stable at 1-year follow-up (Figure 1).

## Pathology

**Figure 2:**
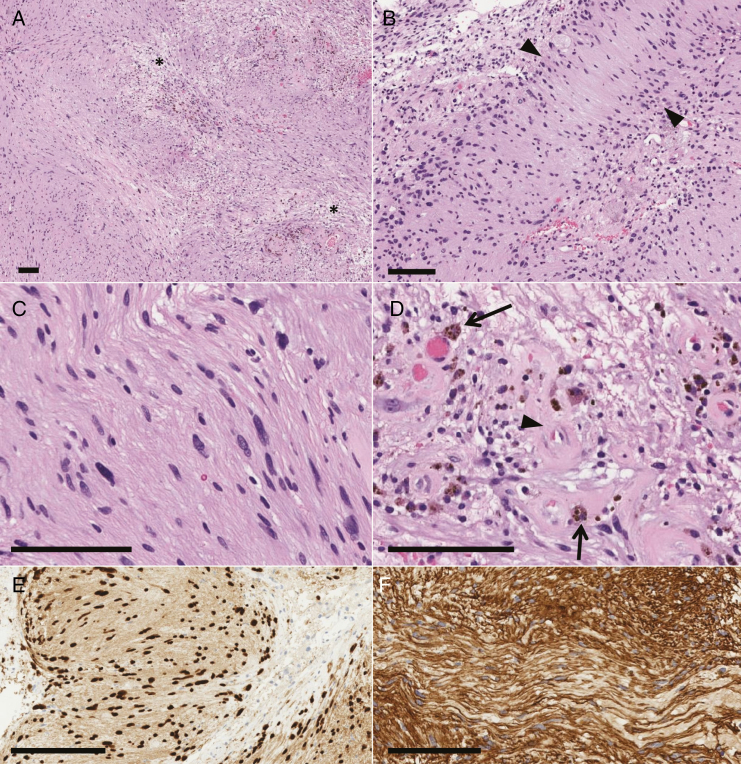
Pathological findings. (A) The tumor was composed of cells arranged in alternating dense fascicles and loose hypocellular areas (asterisks). (B) Areas of vague nuclear palisading were occasionally seen (arrowheads). (C) Tumor cells showed elongated nuclei with mild degenerative atypia. (D) Hyalinized vasculature (arrowhead) and perivascular hemosiderin-laden macrophages (arrows) were identified. (E) On immunohistochemistry, the tumor showed diffuse nuclear staining with SOX10 and (F) diffuse pericellular staining with collagen IV. Scale bars = 100 µm.

Histopathology showed a spindle cell neoplasm with alternating dense interweaving fascicles and looser hypocellular areas (Figure 2A). There were occasional areas of vague nuclear palisading (Figure 2B). Tumor cells had elongated nuclei with mild degenerative atypia (Figure 2C). There was no mitotic activity or necrosis. Vasculature within the tumor was markedly hyalinized and there were abundant perivascular hemosiderin-laden macrophages (Figure 2D). Reticulin stain showed dense pericellular wrapping. Immunohistochemistry showed diffuse nuclear expression of SOX10 (Figure 2E) and cytoplasmic expression of GFAP. EMA, CD34, and neurofilament were negative. Diffuse pericellular staining was seen with collagen IV (Figure 2F). MIB1 labeling index was approximately 1–2%. By histology, the above was in keeping with a WHO grade-I schwannoma.

## Molecular Diagnostics

**Figure 3.**
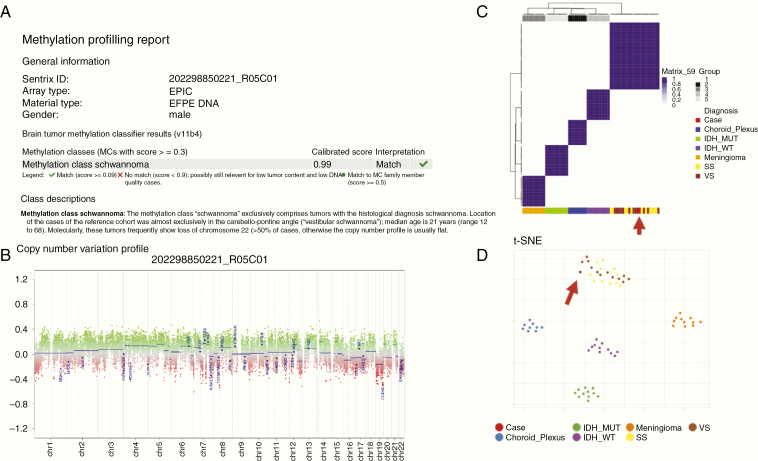
Results of DNA methylation profiling analysis. (A) The top score from the DKFZ classifier v11b4 (Molecular Neuropathology, Heidelberg, Germany) suggests methylation class schwannoma as the most likely diagnosis based on methylation signature. (B) Chromosomal copy number variation profile generated from raw methylation data did not demonstrate any large-scale alterations, in keeping with schwannoma. (C and D) Unsupervised hierarchical clustering approach demonstrates that the tumor sample most closely clustered with schwannomas (vestibular and spinal) when strictly compared to meningiomas, gliomas, and choroid plexus tumors included. IDH_MUT, isocitrate dehydrogenase mutant; IDH_WT, isocitrate dehydrogenase wildtype; SS, spinal schwannoma; VS, vestibular schwannoma.

Given the unusual location for a schwannoma, molecular testing of the tumor was sought for confirmation of the diagnosis. Eight unstained slides of paraffin-embedded tissue were used as samples and DNA was extracted and subjected to DNA methylation profiling (Infinium MethylationEPIC Kit) as per the manufacturer’s protocol. Raw methylation data were subjected to classification using a recently developed and validated methylome-based CNS tumor classification model.^3^ The top-ranked diagnosis was schwannoma (Figure 3A). Chromosomal copy number variation profile generated from raw methylation data did not demonstrate any large-scale alterations, in keeping with the diagnosis of schwannoma as opposed to other pathologies in the differential diagnosis (Figure 3B). Loss of chromosome 22 was not identified. Using unsupervised hierarchical clustering approaches, the tumor sample most closely resembled schwannomas when strictly compared to other tumors in the differential diagnosis (meningiomas, gliomas, and choroid plexus tumors; Figure 3C and D). Taken together, the above molecular analysis provided confirmation of the histological diagnosis of schwannoma.

## Discussion

Intraventricular schwannomas are rare entities with only 31 prior cases reported worldwide.^2^ The majority are located in the lateral ventricles (63.6%) with the rest in the fourth (27.3%) and the third (9.1%) ventricles. There have been 2 reported cases of “malignant schwannomas” that recurred early after subtotal resection with intracranial metastasis despite adjuvant radiotherapy.^4,5^ For benign intraventricular schwannomas, total resection was curative and was achieved in 25 of the 31 cases. Even those with a subtotal resection did not demonstrate any recurrence at 10 years, suggesting close radiographic surveillance may be appropriate if the pathology does not demonstrate malignancy.^2,6^

There are several theories regarding the pathogenesis of intraventricular schwannomas. Benedikt^7^ described the association of Schwann cells with peripheral or autonomic nerve fibers located within the choroid plexus of the ventricle. These Schwann cells can undergo a neoplastic transformation and become benign or malignant schwannomas. Another theory suggests that ectopic debris from neural crest cells that migrate into the ventricular system during disorganized or abnormal embryogenesis can also undergo neoplastic transformation.^8^ These theories may explain the close anatomic association of these tumors with choroid plexus in several previous reports, as well as in our own case. A final theory suggests that pluripotent mesenchymal stem cells from the subventricular zone may differentiate into Schwann cells following tissue injury, such as in multiple sclerosis, post-stroke, and other pathologies.^9,11^

All previous diagnoses of intraventricular schwannomas have relied entirely on histology and immunohistochemistry. Recent revisions of the WHO classification in 2016 integrate molecular alterations with histopathological changes for specific tumors. For schwannomas, the diagnosis remains entirely histopathological. We previously established the molecular landscape of schwannomas and have shown that DNA methylation profiling identifies schwannoma subgroups based on anatomical location (primarily vestibular versus spinal schwannomas).^12–14^ In our unsupervised analysis, we included known cases of choroid plexus tumors (papillomas and carcinomas), gliomas (IDH-mutant and wildtype), meningiomas, and schwannomas (cranial/vestibular and peripheral) to determine the similarity of this case’s methylation profile to known entities. Overall, although our analysis included relatively few cases of cranial versus peripheral schwannomas compared to previous studies, we found this tumor most closely resembled tumors of vestibular origin. However, future studies are needed to determine if intraventricular schwannomas represent a new methylation subgroup with different tumor biology and mutational landscape.

Our case demonstrates the clinical utility of DNA methylation profiling in brain tumor classification and highlights how epigenome-wide DNA methylation analysis can be leveraged as an additional tool to improve brain tumor classification using modeling approaches, particularly for challenging brain tumors in rare locations, with discriminative capabilities reaching 99%.^3,15^

## Funding

No funding was allocated for this work.

## Authors’ Contributions

J.Z.W., N.P., N.I., C.V., S.S., A.G., P.D., and F.N. prepared the text and figures. F.N., S.K., and M.Y. provided the methylation data. J.Z.W., F.N., S.S., N.P., N.I., and G.Z. edited the clinical portions.
